# Endothelium-Derived Netrin-4 Supports Pancreatic Epithelial Cell Adhesion and Differentiation through Integrins α2β1 and α3β1

**DOI:** 10.1371/journal.pone.0022750

**Published:** 2011-07-29

**Authors:** Mayra Yebra, Giuseppe R. Diaferia, Anthony M. P. Montgomery, Thomas Kaido, William J. Brunken, Manuel Koch, Gary Hardiman, Laura Crisa, Vincenzo Cirulli

**Affiliations:** 1 Department of Pediatrics, University of California San Diego, La Jolla, California, United States of America; 2 Department of Anatomy and Cellular Biology, State University of New York, Downstate Medical Center, Brooklyn, New York, United States of America; 3 Center for Biochemistry, Institute for Oral and Musculoskeletal Biology, University of Cologne, Cologne, Germany; 4 Biomedical Genomics Microarray Facility (BIOGEM), University of California San Diego, La Jolla, California, United States of America; 5 Department of Medicine, University of California San Diego, La Jolla, California, United States of America; 6 Department of Molecular and Experimental Medicine, The Scripps Research Institute, La Jolla, California, United States of America; 7 Department of Medicine, University of Washington, Seattle, Washington, United States of America; 8 Department of Pharmacology, University of Washington, Seattle, Washington, United States of America; 9 Institute for Stem Cell and Regenerative Medicine, University of Washington, Seattle, Washington, United States of America; University of Bremen, Germany

## Abstract

**Background:**

Netrins have been extensively studied in the developing central nervous system as pathfinding guidance cues, and more recently in non-neural tissues where they mediate cell adhesion, migration and differentiation. Netrin-4, a distant relative of Netrins 1–3, has been proposed to affect cell fate determination in developing epithelia, though receptors mediating these functions have yet to be identified.

**Methodology/Principal Findings:**

Using human embryonic pancreatic cells as a model of developing epithelium, here we report that Netrin-4 is abundantly expressed in vascular endothelial cells and pancreatic ductal cells, and supports epithelial cell adhesion through integrins α2β1and α3β1. Interestingly, we find that Netrin-4 recognition by embryonic pancreatic cells through integrins α2β1 and α3β1 promotes insulin and glucagon gene expression. In addition, full genome microarray analysis revealed that fetal pancreatic cell adhesion to Netrin-4 causes a prominent down-regulation of cyclins and up-regulation of negative regulators of the cell cycle. Consistent with these results, a number of other genes whose activities have been linked to developmental decisions and/or cellular differentiation are up-regulated.

**Conclusions/Significance:**

Given the recognized function of blood vessels in epithelial tissue morphogenesis, our results provide a mechanism by which endothelial-derived Netrin-4 may function as a pro-differentiation cue for adjacent developing pancreatic cell populations expressing adhesion receptors α2β1 and α3β1 integrins.

## Introduction

During development, cells must often migrate great distances through the extracellular environment that provides informational cues for navigation to their target location and functional maturation. Accordingly, proper architectural organization of different cell types within developing tissues arises from tightly controlled mechanisms of cell adhesion, migration, proliferation, differentiation, and survival [1]. These processes all depend on cell interactions with select components of the extracellular matrix (ECMs), through engagement of specific cellular receptors of the integrin family and with cytokines, chemokines and growth factors [2], [3].

The pancreas provides a valuable model system to study organogenesis. It is an epithelial tissue containing endocrine and exocrine cells, a ductal system, a vascular network, sensory and sympathetic innervation, and a stromal component. It develops by a process of branching morphogenesis from the primitive gut epithelium [4], initially depending on the direct contact of the midline endoderm with the notocord [5], [6], followed by the evagination of a dorsal and a ventral bud. Interaction of the epithelial component of these two pancreatic primordia with the ECM, growth factors, and other signalling molecules provided by the surrounding mesenchyme, causes waves of cell proliferation, branching morphogenesis, and differentiation of both the exocrine and endocrine cell lineages [7], [8], [9].

In the developing human pancreas, we have previously reported that Netrin-1 is produced by a discrete population od ductal cells, is deposited in basal membranes, and supports epithelial cell adhesion and migration of PDX-1^+^ pancreatic progenitors through integrin receptors α6β4 and α3β1 [10]. More recently, it has been shown that Netrins provide anti-apoptotic cues to adult β-cells [11]. In the present study we extended our analysis to Netrin-4, another member of the Netrin family whose primary sequence is closely related to the laminin β chains [12], [13], [14].

## Results

**Figure 1:**
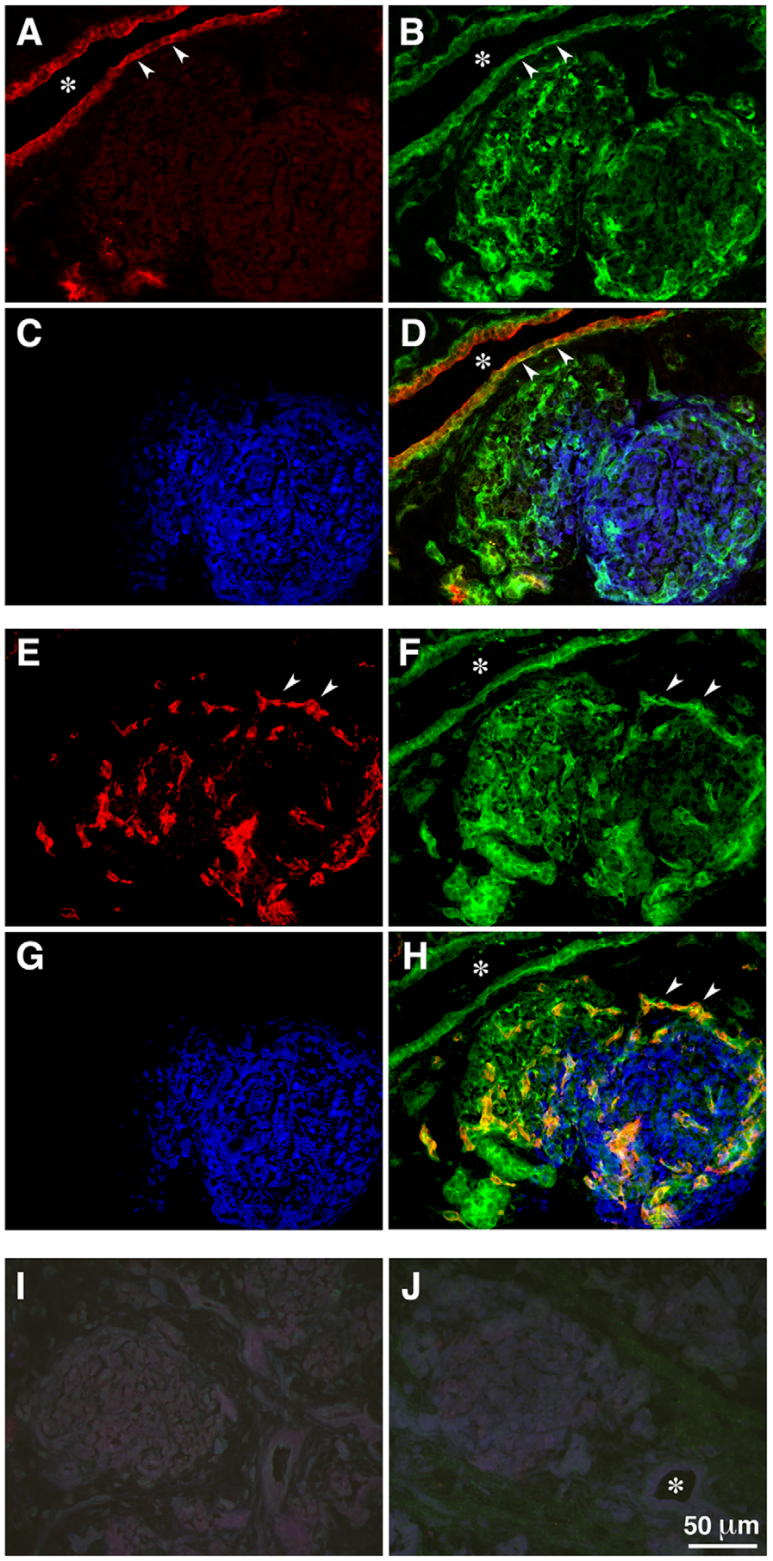
Expression of Netrin-4 in the developing pancreas. Three-color immunofluorescent staining of fetal pancreas showing expression of Netrin-4 (**B**) in ductal cells, identified by the ductal marker CA19-9 (**A**). Arrowheads in **A**, **B**, and **D** points to Netrin-4 immunoreactivity in basal membranes of ductal cells. Insulin-expressing cells (**C**, **G**) do not show Netrin-4-specifc immunoreactivity, although they appear to be infiltrated by strings of Netrin-4-postive cells (**F**, arrowheads) that co-express the endothelial marker PECAM-1 (**E**, arrowheads). Images presented in **A–D** and **E–H** were acquired from consecutive sections. (**I**, **J**) negative controls using isotype matched IgGs. (*) Lumen of ductal structures.

**Figure 2:**
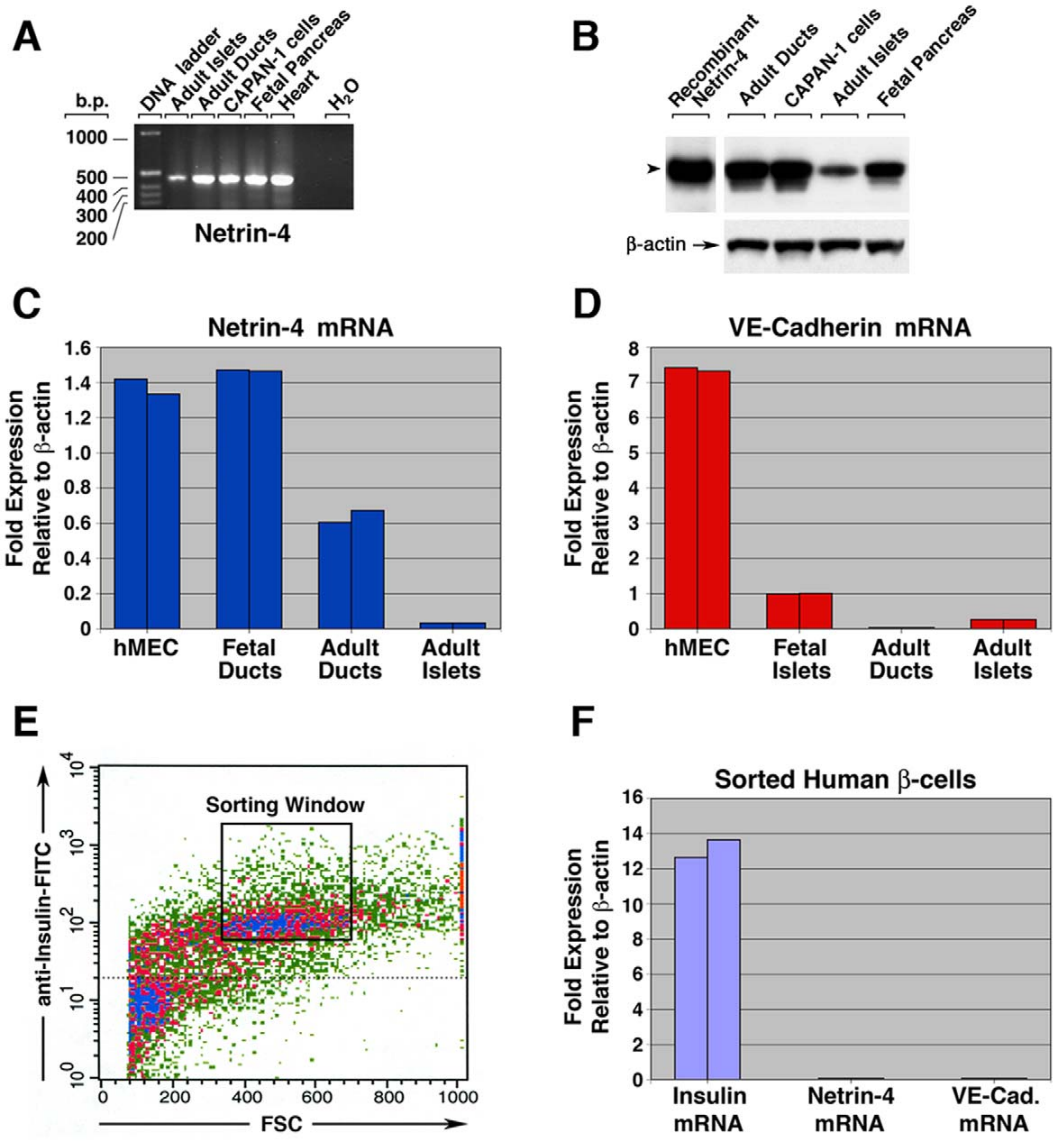
Identification of pancreatic cell types expressing Netrin-4. PCR analysis (**A**) and Western blotting (**B**) on select pancreatic cell populations show significant levels of Netrin-4 expression in adult primary ductal cells, ductal cell line CAPAN-1, and fetal pancreatic cells, and low levels in pancreatic islets. Representative of n = 3. SYBR green qPCR (**C**) for Netrin-4-specific transcripts in primary microvascular endothelial cells (hMEC), fetal and adult pancreatic ductal cells, and intact adult islets. SYBR green qPCR for the endothelial-specific cell adhesion molecule VE-cadherin (**D**) showing that resident endothelia cells are positioned within fetal and adult islets. Fluorescence-activated cell sorting of a single cell suspension from isolated human fetal islets immunostained for insulin (**E**), and SYBR green qPCR analysis for insulin, Netrin-4, and VE-cadherin (**F**). Data presented in C, D, E and F are representative of n = 3, with each SYBR green qPCR reaction performed in duplicate.

In order to evaluate the expression pattern for Netrin-4, we performed three-color immunofluorescence staining in mid gestation human embryonic pancreas. These experiments revealed that Netrin-4-specific immunoreactivity highlights a large number of cells. Immunostaining for the ductal cell marker CA19-9 [15] (Figure 1A), Netrin-4 (Figure 1B), and insulin (Figure 1C), show significant localization of Netrin-4 in ductal structures (Figure 1B and D, arrowheads), and in CA19-9-negative cells that appear to infiltrate the insulin-positive islet cell clusters (Figure 1D). Additional three-color immunostaining for the endothelial marker PECAM-1 (Figure 1E), Netrin-4 (Figure 1F), and insulin (Figure 1D), demonstrates that these string-like structures, strongly positive for Netrin-4, are blood vessels (Figure 1E, F and H, arrowheads). PCR analysis (Figure 2A) and Western blotting (Figure 2B) confirmed high levels of expression of Netrin-4 in ductal cells and low levels in pancreatic islets. Subsequent quantitative analysis of Netrin-4-specific transcripts by SYBR green qPCR revealed that Netrin-4 is readily detected in primary microvascular endothelial cells (hMEC) (Figure 2C), as well as fetal and adult pancreatic ductal cells, whereas intact adult islet clusters only showed very low levels of Netrin-4 transcripts. The low level of Netrin-4 expression detected in adult islet cell clusters (relative to hMEC cells used as a positive control) was found to correlate with low levels of expression of the endothelial-specific cell adhesion molecule VE-cadherin (Figure 2D). These data, therefore, suggested that detection of Netrin-4 by Western blotting in islet samples (Figure 2B) may originate from endothelial cells resident in the islet cell clusters. To investigate this possibility further, a single cell suspension prepared from isolated islets was immunostained for insulin, and islet β-cells purified by fluorescence-activated cell sorting (Figure 2E). SYBR green qPCR analysis of these purified islet β-cells demonstrated that they are devoid of Netrin-4 transcripts (Figure 2F), and thereby further supports the notion that Netrin-4 expression detected in whole islets is derived from resident intra-islet vascular cells.

**Figure 3:**
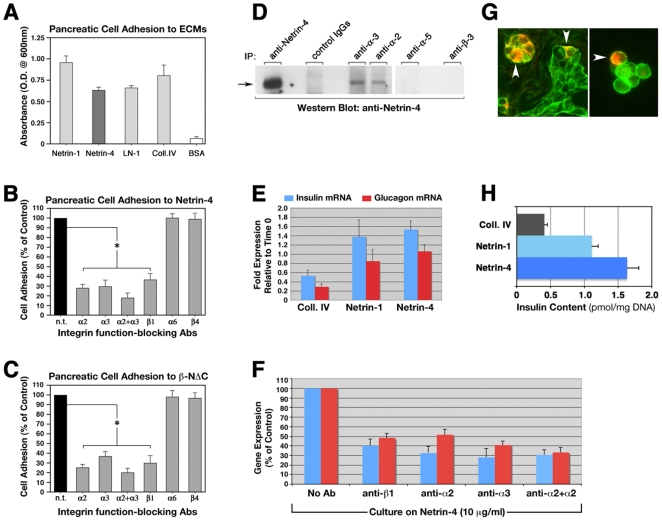
Netrin-4 supports epithelial cell adhesion through integrin receptors and fosters the expression of islet-specific differentiation genes. (**A**) Adhesion of pancreatic epithelial cells to Netrin-1, Netrin-4, LN-1 and Collagen IV. BSA was used as negative control. (**B**) Cell adhesion to Netrin-4 in the absence (n.t., no treatment) or presence of function-blocking antibodies to select integrin subunits. Note the significant blockade of cell attachment to Netrin-4 in the presence of anti-α2, -α3, -β1, or a combination of anti-α2 and anti α3 function-blocking antibodies. (**C**) Similar results were obtained when cells were plated on a modified recombinant Netrin-4 (ΔC-Netrin-4) that lacks 155aa from its carboxy terminal domain. Data in **A** and **B** are representative of n = 4, and in C of n = 3. *p<0.001 ANOVA followed by post-test Bonferroni's multiple comparison test. (**D**) Immunoprecipitation using anti-Netrin-4, -α2, -α3, -α5, -β3 or control IgGs, followed by Western blotting for Netrin-4 revels that α2 and α3, but not α5 or β3, integrin subunits selectively interacts with Netrin-4 in live cells. Representative of n = 3. (**E**) TaqMan PCR analysis for insulin and glucagon mRNAs demonstrates that overnight culture of embryonic pancreatic cells on Netrin-4 promotes the expression of these two islet-specific differentiation genes, when compared to Collagen IV. Culture on Netrin-1, that we reported to engage integrin α3β1 as a receptor [10], also revealed significantly higher levels of insulin- and glucagon-specific transcripts when compared to Collagen IV (n = 6); statistical significance of differences in insulin (p<0.001) and glucagon (p<0.005) expression between Netrins and Coll. IV overnight cultures was determined by ANOVA followed by post-test Bonferroni's multiple comparison test. (**F**) Blockade of α2, α3, β1, or α2 and α3 simultaneously, significantly reduced Netrin-mediated pro-differentiative effects on pancreatic cells (n = 4). (G) Specific immunoreactivity for the α3 integrin subunit (green fluorescence) is detected both *in situ* (G, left panel) and *in vitro* (G, right panel) in insulin-producing cells (red fluorescence, arrowheads). (H) Insulin content measured in embryonic pancreatic cells cultured on either Collagen IV, Netrin-1, or Netrin-4 (n = 4).

Based on the high homology that Netrin-4 shares with the β1 chain of Laminins, a major component of basement membranes providing adhesive and signaling cues to adjacent epithelial cells [12], we investigated whether Netrin-4 could serve an ECM-like function by supporting adhesion of pancreatic epithelial cells. In these experiments, using embryonic pancreatic epithelial cells, we observed that Netrin-4 functions as an efficient substrate for cell adhesion (Figure 3A). Adhesion to Netrin-4 was similar to that detected on Laminin-1 and Collagen IV, and on Netrin-1 that we have previously defined as an adhesive substrate for pancreatic epithelial cells [10]. These results suggest that production of Netrin-4 by intra-islet blood vessel, or by ductal cells, may provide adhesive interactions impacting upon adjacent epithelial cells. In light of the recent demonstration that Netrin-4 is a component of basement membranes, and that it regulates basal membrane assembly [16], our results herein provide functional evidence for an important role of Netrin-4 in the regulation of cell interactions with the extracellular matrix.

Based upon the results described above, we performed experiments designed to disclose the identity of a putative integrin receptor(s) that could mediate epithelial cell adhesion to Netrin-4. To do this, we used a panel of integrin-specific function-blocking antibodies (Figure 3B). These studies revealed that blockade of α2, α3, and β1 integrin subunits significantly inhibited cell adhesion to Netrin-4, indicating that α2β1 and α3β1 heterodimers, two well characterized Laminin receptors [17], [18], are the integrins mediating this adhesive interaction. In support of the specificity of this interaction, blockade of other Laminin receptors such as α6β4 did not affect cell adhesion to Netrin-4 (Figure 3B). Similar results were obtained using a shortened form of recombinant Netrin-4 lacking the C domain (referred to as β-NΔC) [12] (Figure 3C), indicating that integrins α2β1 and α3β1 recognize regions of Netrin-4 away from its carboxy terminal.

To determine whether the observed Netrin-4/integrin interaction also occurs *in vivo*, detergent extracts of embryonic pancreatic explants were immunoprecipitated with either anti-Netrin-4, -α2, -α3, -α5, -β3 antibodies, or control IgGs, and analyzed by Western blotting for Netrin-4-specific immunoreactivity. In these studies, both anti-α2 and anti-α3 antibodies co-immunoprecipitated significant Netrin-4-specific immunoreactivity (Figure 3D), indicating that these two integrins may engage Netrin-4 within an epithelial tissue *in vivo*. Conversely, antibodies specific for α5 and β3 integrin subunits, which are also expressed in the pancreatic epithelium, did not co-immunoprecipitate detectable Netrin-4-specific immunoreactivity (Figure 3D). These results have important functional implications as the engagement of Laminin receptors α2β1 and α3β1 integrins has been linked to cellular differentiation in several tissues [17], [19].

Development of a number of epithelia is tightly dependent on the parallel development of a blood vessel network. In fact, it has been shown that interaction of the endothelium with competent pre-pancreatic endoderm is capable of inducing the development and differentiation of pancreatic islet cells and insulin expression [20]. Thus, consistent with these previous observations, the significant levels of Netrin-4-specific immunoreactivity observed in blood vessels infiltrating insulin-positive developing islet clusters (Figure 1E-H) suggests a function of Netrin-4 in specifying developmental processes associated with pancreatic islet ontogenesis. Therefore, we hypothesized that recognition of Netrin-4 by integrins α2β1 and α3β1 could mediate inductive effects on pancreatic epithelial progenitor cells, and thus foster islet cell differentiation. To test this hypothesis, embryonic pancreatic cells were isolated and allowed to adhere to either purified recombinant Netrin-4, Netrin-1, or to Collagen IV (a major component of basal membranes), and cultured for 18 hours in serum-free conditions, a short-term period that does not support significant cell growth [21]. Cells were then harvested and analyzed by TaqMan PCR for changes in expression of the islet cell-specific differentiation genes insulin and glucagon. The data from these experiments (Figure 3E) indicate that this relatively short-term culture on Netrin-4 is sufficient to significantly increase the expression of both insulin and glucagon, when compared to cells exposed to Collagen type IV (Figure 3E). Culture on Netrin-1, that we reported to also engage integrin α3β1 as a receptor [10], revealed a similar increase of insulin- and glucagon-specific transcripts when compared to Collagen IV (Figure 3E). The observed inductive function of Netrin-4 on insulin expression was also validated at the protein level, as measured by an ultra-sensitive ELISA (Figure 3H).

When function-blocking antibodies to either α2, α3, β1, or to a combination of α2 and α3 integrin subunits, were added to the cells cultured on Netrin-4 a significant reduction of insulin- and glucagon-specific transcripts was detected (Figure 3F), thus providing a mechanistic validation for the pro-differentiative effects of Netrin-4 mediated by the engagement of α2β1 and α3β1 integrins. Supporting evidence for these results is also provided by the observation that the α3 integrin subunit (Figure 3G, green) is expressed by insulin-producing cells *in vivo* (Figure 3G, red, arrowheads), and retained *in vitro* after isolation (Figure 3G, arrowhead).

**Figure 4:**
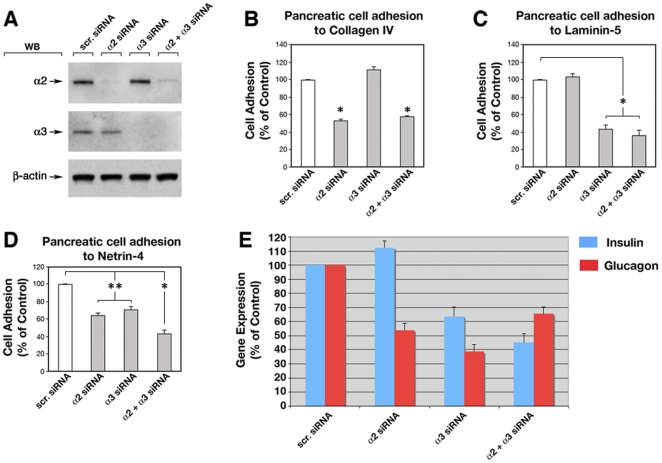
Knock-down of α2 and α3 integrin subunits by siRNA affects pancreatic cell adhesion to Netrin-4 and expression of islet-specific differentiation gene expression. (**A**) Western blot analysis of pancreatic cells transfected with siRNA specific for α2 and α3 integrin subunits effectively knocked-down α2 and α3 protein expression. Down-regulation of α2 and α3 integrin subunits in pancreatic cells resulted in a significant reduction of adhesion to Collagen IV (**B**) Laminin-5 (**C**), and to Netrin-4 (**D**), as well as a decrease in glucagon and/or insulin gene expression when cultured overnight on Netrin-4 (**E**). Data presented are representative of three independent experiments. Data presented in **B**, **C** and **D** are representative of n = 3, *p<0.001 and **p<0.01 as determined by ANOVA followed by post-test Bonferroni's multiple comparison test.

To further validate these results, we transfected cultures of pancreatic epithelial cells with siRNAs specific for α2 and α3 integrin subunits, and assessed the effects of knockdown of these integrins on cell adhesion and on insulin and glucagon gene expression. In these experiments, α2- and α3-specific siRNAs effectively knocked down the expression of α2 and α3 proteins, as determined by Western blotting (Figure 4A). These α2- and α3-deficient pancreatic cells were then tested for cell adhesion to Collagen IV and Laminin-5, used as positive controls for cell adhesion mediated by integrin α2β1 and α3β1, respectively, and to Netrin-4. As shown in Figure 4B–D, α2- and α3-specific siRNAs significantly reduced pancreatic cell adhesion to Netrin-4, as efficiently as to Collagen IV and Laminin-5. The residual cell adhesion to these classical ECM components and to Netrin-4 is likely to be mediated by remnant cell surface expression of α2β1 and α3β1 integrins following knockdown by α2- and α3-specific siRNAs, and/or by alternative receptors such as UNC5A-D also expressed by the pancreatic epithelium (not shown), and reported to interact with this neural chemoattractant [22]. Similarly to the antibody blocking experiments (Figure 3F), siRNA-mediated simultaneous down-regulation of α2 and α3, or of α3 alone decreased both insulin and glucagon gene expression (Figure 4E). In contrast, siRNA-mediated down-regulation of α2 integrin alone solely affected glucagon, but not insulin expression (Figure 4E). This latter result indicates that functional blockade of α2 integrin expressed at the cell surface or interference of its translation by siRNA has differential effects on insulin gene expression.

**Figure 5:**
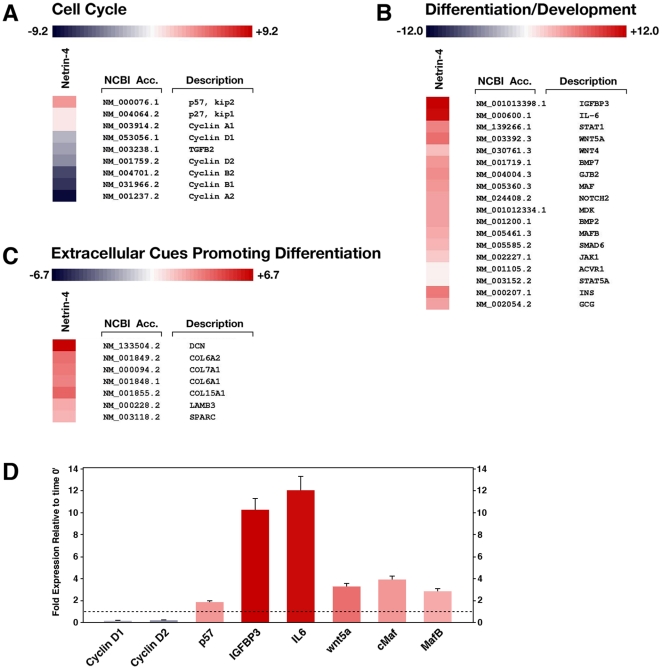
Pancreatic cell adhesion to Netrin-4 promotes cell cycle exit and fosters the expression of pro-differentiation genes. Heatmap of select genes that are either down-regulated (**A**) or up-regulated (**B** and **C**) by an 18-hours exposure of fetal pancreatic cells to Netrin-4. Data are presented as fold increase over time 0′. Note that known negative regulators of the cell cycle such as p57/kip2 and p27/kip1 are up-regulated (**A**), whereas positive regulators such as cyclins are down-regulated (**A**). Conversely, a number of genes whose function has been linked to events of cellular differentiation are all up-regulated (**B**, **C**). Changes in expression of select genes exemplified in panels **A** and **B** were validated by qPCR (**D**), where a value of 1 is equal to no change in gene expression. Complete array data have been deposited in the EBI Array Express Database (accession number pending). Data presented in **D** are representative of two independent experiments.

Finally, to investigate the effects of Netrin-4 on multiple gene networks in fetal pancreatic cells we performed a full genome microarray analysis using the Illumina Human-6v2 Expression BeadChips assay. Using this approach we observed that an 18-hours exposure of fetal pancreatic cells to Netrin-4 affected the expression of multiple genes. As shown in Figure 5, one of the most prominent effects observed is a significant down-regulation of known positive regulators of the cell cycle (cyclins), and up-regulation of negative regulators of the cell cycle such as p57 and p27 (Figure 5A) [23], [24], [25]. Consistent with these effects, we observed not only significant up-regulation of insulin and glucagon (Figure 5B), but also of a number of genes whose function has been directly or indirectly linked to cell fate developmental decisions and/or events of cellular differentiation (Figure 5B–C). These included WNT5, whose function has been linked to events of islet cell migration and development [26], MafB, whose activity has been demonstrated to directly control islet β-cell maturation [27], and Notch2, known to modulate the maintenance of immature cell plasticity and consequently prevent excessive cell differentiation during islet development [28]. The observed changes in expression of several selected genes were validated by qPCR (Figure 5D) and shown to correlate very closely with the microarray results. Together, these results support a pro-differentiation function of Netrin-4 on developmental decisions that the embryonic pancreatic epithelium can adopt upon interaction with this neural chemotropic factor.

## Discussion

Netrins have been extensively studied for their pathfinding guidance properties on select cell populations and axons in the developing central nervous system (CNS) [29]. However, it has been demonstrated that some members of the Netrin family are also expressed outside the CNS where they mediate cell adhesion, migration and differentiation [10], [30], [31], [32].

In this study we report that in the developing human pancreas vascular and ductal cells produce Netrin-4. Consistent with its high homology to the β1 chain of laminins, we find that Netrin-4 functions as an ECM and supports efficient pancreatic epithelial cell adhesion comparable to that supported by classical ECM components such as Laminin-1 and Collagen IV. Based on the recent demonstration that Netrin-4 participates in processes of basal membrane assembly through its interaction with laminin γ1 and γ3 short arms [16], our findings have important implications for the developmental biology of the pancreatic epithelium. In accordance with the well known function of basal membranes in tissue morphogenesis and homeostasis, we demonstrate that pancreatic epithelial cells adhere very efficiently to Netrin-4, and that this interaction is mediated by integrin receptors α2β1 and α3β1, two well characterized laminin receptors. Interestingly, Netrin-4 recognition by developing pancreatic epithelial cells provides differentiation cues, as it elicits significant induction of insulin and glucagon gene expression, thus demonstrating a crucial role of this neural chemotropic factor in the development and/or maintenance of endocrine cell phenotype.

The basal membrane provides informational cues to adjacent cells as it imparts functions through engagement of specific integrin receptors that in turn activate select signaling pathways, ultimately affecting cell fate and behavior [1], [2], [3]. Our identification of Netrin-4 production by blood vessels infiltrating developing islet clusters, together with the recent demonstration that Netrin-4 functions as a structural component of basal membranes through engagement with the γ1 and γ3 short arms of laminin [16], and formation of a protein complex with select laminin receptors [33], holds important functional consequences. Accordingly, we demonstrate that presentation of Netrin-4 to pancreatic epithelial cells supports attachment through specific integrin receptors (i.e., α2β1 and α3β1). Based on the high functional redundancy of select integrin receptors, and on the high homology of Netrin-4 to the laminin β chains [12], [13], [14], our prediction that pancreatic epithelial cells expressing α2β1 and α3β1 integrins would use these laminin receptors to recognize Netrin-4 proved correct, further validating the role of Netrins as crucial components of the extracellular microenvironment that can affect cellular behavior [10], [34].

The demonstration that pancreatic epithelial cells adhere to Netrin-4 using laminin receptors α2β1 and α3β1 integrins adds important insights into the mechanisms of cell-cell cross-talk that controls pancreatic cell differentiation. Thus, while evidence has been provided for laminins to play important functions in pancreatic islet cell development [7], [35], [36], our identification of endothelium-derived Netrin-4 as an additional ligand for α2β1 and α3β1 integrins that can affect islet cell phenotype provides a more complete picture of the complex hierarchy of interactions that govern islet cell responses to the extracellular microenvironment. The implications of our studies extend far beyond the developmental biology of pancreatic islets. Thus, it should be recalled that engagement of Laminin receptors α2β1 and α3β1 integrins has been linked to events of cellular differentiation in several other tissues [17], [18]. For example, signaling through α2 and α3 integrins has been shown to affect the differentiation of neural cell types [17], [18], epithelial cells of the intestine and salivary gland [37]–[38], and osteoblastic differentiation of bone marrow cells [39]. Hence, our observations provide original evidence for an important function of α2β1 and α3β1 as pro-differentiative integrin receptors in developing islet cells. These two receptors are of particular interest when compared to other integrins shown to mediate significant down-regulation of islet hormones expression over time [40], [41], [42]. Intriguingly, we found that while function-blocking antibodies to cell surface α2 integrin down-regulated both insulin and glucagon gene transcription, knockdown of α2 integrin by siRNA only affected glucagon expression. This discrepancy may be explained by the recent observation that knockdown of the α2 integrin can modify the expression of other integrin subunits, and/or activate the utilization of alternative integrin heterodimers [43]. In turn, such effects on the modulation of other integrins may affect insulin gene expression.

One of the most important functional consequences of the interaction of the pancreatic epithelium with Netrin-4 is the induction and/or maintenance of islet differentiation traits. These results suggest that Netrin-4 may be one of the endothelial-derived factors postulated to exhibit inductive functions on the pancreatic epithelium [35], [44]. Of further interest are the inductive cues that Netrin-4 exerts on pancreatic cells, as uncovered by our genome wide gene array. Results of these experiments clearly demonstrate that exposure of fetal pancreatic epithelial cells to Netrin-4 affects the expression of many genes involved in the control of the cell cycle. Thus, we observed a significant down-regulation of positive regulators of the cell cycle (e.g., cyclins), and up-regulation of negative regulators of the cell proliferation (e.g., the cyclin-dependent kinase inhibitor p57^kip2^) [45], [46]. This result is of particular interest especially in light of the demonstration that p57^kip2^ is expressed in human pancreatic β-cells [47] and it has been shown to mediate cell cycle exit of pancreatic progenitors [25].

Among other genes whose expression has been directly or indirectly linked to events of cellular differentiation, we find significant up-regulation of insulin-like growth factor binding protein-3 (IGFBP-3), reported to affect differentiation of colon epithelial cells [48] and mammary epithelial cells [49]; c-Maf and MafB, reported to regulate insulin gene transcription and islet β-cell maturation [27], [50]; and bone morphogenetic protein-2 (BMP-2), previously implicated in the endocrine differentiation of pancreatic acinar-like cells [51], as well as in the differentiation of smooth muscle cells and osteoblasts [52], [53], [54].

Another gene induced by Netrin-4 is IL-6. This multifunctional cytokine has been shown to induce astrocyte differentiation from neuroepithelial cells [55] and to increase insulin message and secretion in hamster β-cells [56]. The induction of WNT5a is also of interest since it has been directly linked to events of islet cell migration and development [26], and reported to promote axon differentiation of hippocampal neurons [57], as well as differentiation of neural progenitors [58].

A number of genes encoding ECM proteins are also induced in cells cultured on Netrin-4. Among these, decorin, an ECM proteoglycan, has been shown to promote muscle cell differentiation [59], [60], and mammary cell differentiation [61]. The alpha2 chain of collagen type VI is also induced by Netrin-4. Its expression has been shown to be up-regulated during adipocyte and chondrocyte differentiation [62], [63].

Collectively, our studies identify integrins α2β1 and α3β1 as receptors for Netrin-4, and provide evidence for a novel function of this neural chemotropic factor as an extracellular cue that supports epithelial cell adhesion and fosters cellular differentiation. Based on the reported expression of Netrin-4 and integrins α2β1 and α3β1 in a number of cell types [12], [14], [17], [18], [19], [64], [65], [66], the results of our studies have important implications for the ontogeny and homeostasis of various tissues.

## Materials and Methods

### Ethics Statement

Human pancreatic tissue specimens were obtained from Advanced Biomedical Research (ABR, Alameda, CA), and from The Laboratory of Developmental Biology (University of Washington, Seattle, WA). Human tissues were provided as “pre-existing pathological specimens” (i.e., not through the recruitment of living human subjects), and written consent for tissue donation was obtained by the procurement entities. All studies described in this manuscript were reviewed and approved by the Human Research Protections Programs of University of California San Diego (La Jolla, CA), and the University of Washington (Seattle, WA).

### Immunofluorescence and confocal microscopy

Two and three color immunofluorescence and confocal analysis were performed on cryostat 7 µm sections of fetal pancreata, or isolated embryonic pancreatic cells, as previously described [10]. Primary antibodies used: anti-Netrin-4 polyclonal R33 and monoclonals 61 and 9F11 [12]; anti-α3 (P1B5), anti-α6 (GoH3), anti-β1 (LM534) (Chemicon); anti-β4, UMA9 (Ancell Corp.), anti-insulin (The Binding Site); anti-glucagon (Sigma); anti–platelet endothelial cell adhesion molecule-1 (PECAM-1) (Santa Cruz Biotechnology). Fluorophore-labeled F(ab)_2_ secondary antibodies preadsorbed on multiple species serum proteins (Jackson ImmunoResearch) were: LissamineRhodamine (LRSC)-donkey anti-rabbit; Fluorescein Isothiocyanate (FITC)-donkey anti-mouse and donkey anti-rat; Cy5-donkey anti-sheep. Stained sections were viewed on a Zeiss Axiovert 35M microscope equipped with a confocal attachment (MRC-1024, Bio-Rad).

### Cell Culture and Adhesion Assays

Specimens of human fetal pancreatic tissue were obtained from Advanced Biomedical Research (ABR, Alameda, CA). These tissues were provided as “pre-existing pathological specimens” (i.e., no recruitment of living human subjects involved), and all studies described in this report were reviewed and approved by the UCSD Human Research Protections Program. Human pancreatic epithelial cells (at 17–21 weeks gestation) were prepared as previously described [10]. Cultures were expanded on HTB-9-coated dishes for five days in RPMI-1640 containing 10% FCS, antibiotics, and supplemented with 10 ng/ml recombinant human hepatocyte growth factor/scatter factor (rhHGF/SF; a generous gift of Genentech, San Francisco, CA) [10]. Expanded cells (70–80% confluent) were then used for adhesion assays in 96-well high binding EIA plates (Costar, Corning, NY) coated with 10 µg/ml of Netrin-1, Netrin-4, Laminin-1, or Collagen type IV overnight at 4°C, and blocked with 5% BSA for 60 minutes prior to use.

Fetal and adult pancreatic ductal cells were isolated by hand picking of ductal structures from collagenase (Liberase; Roche) digests that are readily recognizable and distinguishable from acinar and islet clusters under a stereo-microscope. Ductal structures were then used for analysis of Netrin-4 expression by either PCR or Western blotting. Production of recombinant Netrin-1 and Netrin-4 has been previously described [10], [12]. Laminin-1 and Collagen type IV were purchased from BD Biosciences. Function-blocking mAbs to integrins α2 (P1E6), α3 (P1B5), α6 (GoH3), β1 (P4C10), and αvβ3 (LM609) were from Chemicon. Rabbit anti-β4 (R5710) was kindly provided by Dr. Vito Quaranta (Vanderbilt University). Cells were resuspended in serum-free fibroblast basal medium (Cambrex BioScience) supplemented with 0.5% BSA, and 0.4 mM MnCl_2_, seeded in triplicate wells (5×10^4^ cells/well), and allowed to adhere to the various matrices for 90 minutes. Treatment with integrin function-blocking antibodies (40 µg/ml) was for 30 minutes prior to plating. After washing of non-adherent cells, adherent cells were fixed in 3% paraformaldehyde and stained with toluidine blue in 1% sodium borate. Adherent cells were quantified by counting the number of stained cells. Alternatively, the dye was eluted with 10% acetic acid and the absorbance was measured in a spectrophotometer at 600 nm.

### Nucleofection

Human fetal pancreatic cells were expanded for five days on HTB-9-coated dishes in the presence of HGF/SF as described [7], [10]. These culture conditions resulted in >95% undifferentiated epithelial cell populations as determined by staining for Ep-CAM [7], [10]. Cells were subsequently harvested and transfected with the Amaxa Nucleofection system according to the manufacturer's instructions. For each transfection, the cell pellet was resuspended in 100 µl of Amaxa nucleofector solution from the Basic Nucleofector Kit for primary mammalian epithelial cells (#VPI-1005; Amaxa GmbH), mixed with 30 nM of *Silencer* Validated α2 siRNA (ID# 106725), *Silencer* Pre-designed α3 siRNA (ID# 111569), or scrambled siRNA (ID# 4611) from Ambion (Austin, TX) at room temperature, and immediately transferred to the Amaxa cuvette and nucleofected using the S-005 program on the Amaxa nucleofector apparatus. By this procedure ∼65% of the cells were successfully transfected, as determined in pilot experiments using the fluorescent reporter (pmaxGFP vector; Amaxa). After transfection, the cells were transferred to 60-mm HTB9-coated dishes containing 5 ml of pre-warmed complete RPMI-1640, and 10 ng/ml HGF and cultured for an additional two days prior to use for biochemical analysis.

### Immunoprecipitation, and Immunoblotting

Fetal or adult pancreatic pancreatic cell preparations, or CAPAN-1 ductal cells (American Type Culture Collection) were lysed for 30 min on ice in 25 mM Hepes, pH 7.5, 150 mM NaCl, 5 mM MgCl_2_,1% Brij 96 (polyoxyethylene-10-oleoyl ether; Sigma-Aldrich) with 1 mM phenylmethylsulfonyl fluoride, 2 mM sodium fluoride, 1 mM sodium orthovanadate, and Complete EDTA-free protease inhibitor cocktail tablets (Roche). The lysates were clarified by centrifugation at 14,000 rpm for 20 min and total proteins were measured using the BCA protein assay (Pierce, Rockford, IL). Proteins (700 µg) were precleared with protein A/G PLUS agarose beads (Santa Cruz Biotechnology) and then incubated with either anti-netrin-4 rabbit polyclonal (KR2), anti-α2 integrin rabbit polyclonal, anti-α3 integrin rabbit polyclonal, anti-α5 integrin rabbit polyclonal, anti-β3 integrin rabbit polyclonal (Chemicon), or rabbit IgG at 4°C overnight. Immune complexes were captured with protein A/G PLUS agarose beads for 3hr at 4°C, washed twice with lysis buffer and three times with PBS containing 0.5% Tween-20 (Sigma-Aldrich), eluted from the beads by boiling in reducing Laemmli buffer, and resolved on a 4%–12% SDS-PAGE gel.

For Western blotting analysis of Netrin-4 expression in pancreatic cells, 10 µg of each cell lysate were loaded per lane. Proteins were transferred to a PVDF membrane, blocked, and incubated with a mouse monoclonal antibody to Netrin-4 (mAb 61 [12] diluted 1∶1000, followed by an HRP-conjugated secondary antibody, and then visualized with ECL Plus chemiluminescence reagent (Amersham Biosciences).

### Cell Sorting

Human fetal islets were isolated by collagenease digestion as previously described [67]. The islet clusters were first dissociated into a free cell suspension, fixed with 1% PFA in PBS for 10 minutes at 4°C, permeabilised with 0.05% Triton X-100 for 10 minutes, non-specific binding blocked in PBS containing 1% BSA, 2% donkey serum, 50 mM Glycine, and 0.5% cold water fish gelatin. After washing in FACS buffer (HBSS, 0.1% BSA), the cell suspension was incubated with a guinea pig anti-insulin antibody (ab7842; Abcam) for 30 minutes at 4°C, washed and then reacted with a FITC-conjugated donkey anti-guinea pig antibody (F(ab')2 fragment; Jackson ImmunoResearch Labs). The cell suspension was then washed and resuspended at 1×10^6^ cells/ml and analyzed for sorting of FITC-labeled β-cells using a FACSVantage (Becton Dickinson). Immediately after sorting, β-cells were processed for total RNA isolation using the RecoverAll Total Nucleic Acid Isolation Kit (Applied Biosystems) that is designed to extract total nucleic acids from formalin or paraformalin-fixed samples.

### Analysis of Insulin and Glucagon mRNA expression by Real-time RT-PCR

For analysis of insulin and glucagon gene expression, human fetal pancreatic epithelial cells expanded as previously described [7], [10] on extracellular matrix produced by the bladder carcinoma cell line HTB-9 (American Type Culture Collection). Expanded pancreatic epithelial cells (70–80% confluent) were harvested, and resuspended at 5×10^5^ cells/ml in Opti-MEM I (Invitrogen) supplemented with penicillin-streptomycin, 2 mM GlutaMAX-1 (Invitrogen), and 0.4 mM MnCl_2_. Cells were then seeded and kept in culture for 18 hours in 6-well plates (3 ml/well) that had been previously coated with 20 mg/ml of either collagen type IV, netrin-1, or netrin-4. Total RNA was extracted using TRIzol Reagent (Life Technologies). An aliquot of 7.5×10^5^ cells immediately lysed in TRIzol prior to plating on the various matrices was used as “time 0”. 2.5 mg of total RNA from each sample was treated with RQ1 DNase (Promega), then retrotranscribed using Oligo dT and Superscript II (Invitrogen) following the manufacturer's recommendations.

Real time PCR analysis for quantitative assessment of target genes expression was performed using the ABI Prism 7900HT (Applied Biosystem). SYBR green qPCR results were analyzed by absolute quantification using the following primers: *beta-actin* 5′-CTAAGGCCAACCGTGAAAAGAT-3′, 5′-CACAGCCTGGATGGCTACGT-3′; *VE-Cadherin* 5′-GGCAAGATCAAGTCAAGCGTG-3′, 5′-ACGTCTCCTGTCTCTGCATCG-3′; *Insulin* 5′-CTCACACCTGGTGGAAGCTC-3′, 5′-AGAGGGAGCAGATGCTGGTA-3′; *Netrin-4* 5′-GTCAAGGCCCCAGGAACATTCCAC-3′, 5′-CCTGTCACAACGTCGCCCTGCCA-3′.

Taqman qPCR results were analyzed by relative quantification (“ΔΔ-Ct method”) using the following Taqman gene expression assays (Applied Biosystem): *insulin, Hs_00355773; Glucagon, Hs_00174967_m1; GAPDH, Hs_99999905; RPS16, Hs_01598518_gH.*

### Immunoassay for quantitative determination of human insulin

Insulin protein levels in samples of human pancreatic epithelial cells cultured overnight on either Collagen IV, Netrin-1, or Netrin-4, were measured using an ultrasensitive enzyme-linked

immunosorbent assay (ELISA) (Alpco Diagnostics, Windham, NH). Cells were harvested from the various culture conditions and processed for determination of insulin and DNA content as previously described [40], [41], [42].

### DNA Microarray

Biotinylated cRNA was prepared using the Illumina RNA Amplification Kit, Catalog #1L1791 (Ambion, Inc., Austin, TX) according to the manufacturer's directions starting with 250 ng total RNA. The labeling approach utilizes a modified Eberwine protocol [68], [69] in which messenger RNA is converted to cDNA, followed by an amplification/labeling step mediated by T7 DNA polymerase. The cDNA and cRNA filter cartridges (Ambion) were used according to the manufacturer's instructions for RT and IVT cleanup, respectively. For microarray analysis, the Illumina Human Expression BeadChip was used (Illumina, San Diego). Hybridization of labeled cRNA to the BeadChip, and washing and scanning were performed according to the Illumina BeadStation 500x manual. Essentially, the amplified biotin-labeled human cRNA samples were resuspended in a solution of Hyb E1 buffer (Illumina) and 25% (v/v) formamide at a final concentration of 25 ng/mL. 1.5 mg of each cRNA were hybridized. Hybridization was allowed to proceed at 55°C, for 18 hours after which, the bead array matrix was washed for 10 minutes with 1X High temperature buffer (Illumina), followed by a subsequent 10 minute wash in Wash E1BC buffer. The arrays were then washed with 100% ethanol for 10 min to strip off any remaining adhesive on the chip. A 2 min E1BC wash was performed to remove residual ethanol. The arrays were blocked for 5 minutes with 1% (w/v) casein-PBS, (Pierce). The array signal was developed via 10 min incubation with Streptavidin-Cy3 at a final concentration of 1 mg/mL solution of (GE Healthcare) in 1% casein-PBS blocking solution. The Human Expression BeadChip was washed a final time in Wash E1BC buffer for five minutes and subsequently dried via centrifugation for 4 minutes at a setting of 275 rcf. The arrays were scanned on the Illumina BeadArray Reader, a confocal-type imaging system with 532 (cye3) nm laser illumination. Preliminary data analysis and QC was carried out using the BeadStudio software (Illumina). Simultaneous normalization of multiple microarrays was done using the “*mloess*” method [70]. Data presented are MIAME compliant and raw data have been deposited in the EBI Array Express Database (accession number: E-TABM-867).

### Statistics

Wherever appropriate, statistical significance of differences in data values was validated by analysis of variance (ANOVA), followed by Bonferroni's Multiple Comparison Test, using the Prism-4 statistical package (Graph Pad Software, San Diego, CA), with significance limit set at p<0.05.
